# MnTnHex-2-PyP^5+^, Coupled to Radiation, Suppresses Metastasis of 4T1 and MDA-MB-231 Breast Cancer via AKT/Snail/EMT Pathways

**DOI:** 10.3390/antiox10111769

**Published:** 2021-11-05

**Authors:** Sung-Won Shin, Changhoon Choi, Hakyoung Kim, Yeeun Kim, Sohee Park, Shin-Yeong Kim, Ines Batinic-Haberle, Won Park

**Affiliations:** 1 Department of Radiation Oncology, Samsung Medical Center, Seoul 135710, Korea; camuserik@gmail.com (S.-W.S.); changhoon1.choi@samsung.com (C.C.); yeeun17.kim@sbri.co.kr (Y.K.); Springu.park@sbri.co.kr (S.P.); syeong.kim@sbri.co.kr (S.-Y.K.); 2 Department of Radiation Oncology, Sungkyunkwan University School of Medicine, Seoul 06355, Korea; 3 Department of Radiation Oncology, Korea University Guro Hospital, Korea University College of Medicine, Seoul 02841, Korea; khk614@gmail.com; 4 Department of Radiation Oncology, Duke University School of Medicine, Durham, NC 27710, USA

**Keywords:** manganese porphyrin, MnTnHex-2-PyP^5+^, SOD mimics, radiation therapy, metastasis, NRF2, EMT, adjuvant therapy

## Abstract

Tumor migration and invasion induced by the epithelial-to-mesenchymal transition (EMT) are prerequisites for metastasis. Here, we investigated the inhibitory effect of a mimic of superoxide dismutase (SOD), cationic Mn(III) *ortho*-substituted *N*-n-hexylpyridylporphyrin (MnTnHex-2-PyP^5+^, MnHex) on the metastasis of breast cancer in cellular and animal models, focusing on the migration of tumor cells and the factors that modulate this behavior. Wound healing and Transwell migration assays revealed that the migration of mouse mammary carcinoma 4T1 cells was markedly reduced during the concurrent treatment of MnHex and radiation therapy (RT) compared with that of the control and RT alone. Bioluminescence imaging showed that MnHex/RT co-treatment dramatically reduced lung metastasis of 4T1 cells in mice, compared with the sham control and both single treatments. Western blotting and immunofluorescence showed that MnHex treatment of 4T1 cells reversed the RT-induced EMT via inhibiting AKT/GSK-3β/Snail pathway in vitro, thereby decreasing cell migration and invasion. Consistently, histopathological analyses of 4T1 tumors showed that MnHex/RT reduced Snail expression, blocked EMT, and in turn suppressed metastases. Again, in the human metastatic breast cancer MDA-MB-231 cell line, MnHex inhibited metastatic potential in vitro and in vivo and suppressed the RT-induced Snail expression. In addition to our previous studies showing tumor growth inhibition, this study demonstrated that MnHex carries the ability to minimize the metastatic potential of RT-treated cancers, thus overcoming their radioresistance.

## 1. Introduction

The in vivo redox imbalance between reactive oxygen/nitrogen/sulfur species (ROS/RNS/RSS) and the antioxidant defenses inflicts damage to the redox-based signaling pathway and induces oxidative damage to biological molecules. This is demonstrated in various pathological states such as radiation damage and tumor. Redox imbalance in the tumor is particularly relevant during tumor growth and neovascularization. Cationic Mn(III) *meso*-tetrakis(*N*-n-hexylpyridinium-2-yl)porphyrin (MnTnHex-2-PyP^5+^;MnHex), a potent lipophilic mimic of the family of porphyrin-based superoxide dismutases (SOD), has been shown to reduce oxidative damage by directly or indirectly reducing the levels of reactive species, thereby restoring the cellular physiological redox state [1,2,3,4,5]. Its analog, MnTnBuOE-2-PyP^5+^ [Mn(III) *meso*-tetrakis(*N*-n-butoxyethylpyridinium-2-yl)porphyrin, BMX-001], is in four clinical trials as radioprotectant of normal tissue and tumor radio- and chemosensitizer [6,7]. Our knowledge of the mechanism of action of Mn porphyrins has matured over the last 10 years. Given its four biologically accessible oxidation states (2, 3, 4, and 5), its reactivity towards numerous low-molecular-weight reactive species and proteins, and the abundant redox-based metabolic pathways of a cell, we are still far away from fully comprehending the biology of Mn porphyrins.

In most solid tumors, radiation therapy (RT) is an effective treatment modality. However, stabilized hypoxia-inducible factor 1-alpha (HIF-1α) upregulates the vascular endothelial growth factor (VEGF), promoting abnormal angiogenesis in the tumor and increasing the resistance of tumor cells to RT. Reactive species (RS), which are produced during tumor irradiation, are involved in the stabilization of HIF-1α and HIF-1α-regulated mRNA [8]. Mn porphyrins (MnPs) reportedly suppress HIF-1α activity [9]. Such an effect likely occurs via MnP-driven inhibition of nuclear factor-кB, NF-кB, as it controls HIF-1α [9,10,11,12,13,14]. MnPs further inhibit the activity of other transcription factors, such as activator protein-1 (AP-1) and specificity protein-1 (SP-1) [15,16,17,18,19,20,21].

A study on MnTnHex-2-PyP^5+^ in the presence of radiation in a 4T1 breast cancer mouse subcutaneous model provided evidence that, in addition to its inhibitory effect on NF-кB, this MnP suppresses the activities of several mitogen-activated protein kinases (MAPKs), c-Jun N-terminal kinase (JNK), extracellular signal-regulated kinase (ERK), p38, and AKT [10]. Such actions of MnTnHex-2-PyP^5+^ attenuated DNA damage repair and triggered a shift from pro-survival pathways to apoptotic cell death, thus inhibiting tumor growth. That study also suggested that MnTnHex-2-PyP^5+^ can act as a potent suppressor of cell migration and thus metastasis to normal tissues. The impact of another MnP analog of similar redox properties (MnTE-2-PyP^5+^) on ERK and NF-кB was also reported [11,12,18].

In a mechanistic study on hematological malignancies, Tome’s lab provided unambiguous evidence that MnTE-2-PyP^5+^, in the presence of H_2_O_2_ and glutathione, GSH, catalyzes the *S*-glutathionylation of cysteines of p50 and p65 subunits of NF-кB, which is followed by the inhibition of this transcription factor and the promotion of apoptosis. The effect is much more pronounced in the presence of dexamethasone [16,17]. Tome’s lab further showed the same is true for complexes I and III whose oxidation-mediated inhibition, and subsequent inactivation suppressed ATP production [17]. Yet, the oxidation and inactivation of normal lymphocytes were not demonstrated [17]. It is important to note that the *S*-glutathionylation is the most frequent in vivo oxidative thiol modification [11,13,16,17,18]. The yield of such oxidative modification depends upon the levels of H_2_O_2_ and Mn porphyrin in cancer cells and tumor tissue. MnTE-2-PyP^5+^ (in the absence of dexamethasone) was able to induce only a low level of protein oxidation; it may be expected that those MnPs that accumulate to much higher levels within cells and tissues, such as lipophilic MnTnHex-2-PyP^5+^, may oxidize protein cysteines to a significant extent even as single drugs, without additional sources of reactive species (such as radiation or chemotherapy).

The impact of MnPs on nuclear factor erythroid 2-related factor 2, NRF2, was reported in non-cancer systems only [14,22]. However, the MnP-driven oxidation of cysteine (Cys288) of the regulatory protein, Kelch-like ECH-associated protein 1 (Keap1) was demonstrated by redox proteomics of 4T1 breast cancer cells treated with MnTE-2-PyP^5+^/ascorbate. Since catalytic, the cycling of MnP with ascorbate produces large amounts of H_2_O_2_ which MnP subsequently employs (along with GSH) to oxidize protein cysteines [13,18]. The oxidation of Keap1 cysteine activates NRF2, which in turn upregulates endogenous antioxidative defenses, such as MnSOD, catalase, glutaredoxins (Grxs), peroxiredoxins Prxs), thioredoxin (Trx), and glutathione-*S*-transferase (GST) [13,18]. Redox proteomics [13,18,23] further provided direct evidence that the mitogen-activated protein kinases (MAPKs), PKC, p38 MAPK, NF-кB, and endogenous antioxidative enzymes were also oxidized by MnTE-2-PyP^5+^/ascorbate. Substantial evidence exists that such oxidative modification results in protein inactivation [13,18]. The most recent review summarizes the effects of Mn porphyrins on molecular pathways in normal and cancer cells [7,11,12,18]. It may be thus hypothesized that the MnP-driven activation of NRF2 via oxidation of Keap1, would not have diminished the anticancer and anti-metastatic effects of Mn porphyrin.

The animal and cellular studies on the impact of MnPs on different cancers, including glioma, breast, and head and neck cancer, as well as hematologic tumors, demonstrated that they act as radioprotectants of normal tissue while radiosensitizing tumors [6,10,13,19,24]. The origin of such, seemingly opposing effects lies in the different redox environments of tumor vs. normal cells (primarily H_2_O_2_ levels) as well as different levels of MnPs in those cells [13,18]. Co-localized, high levels of MnP and H_2_O_2_ in tumors [13,18] result in high levels of oxidized and in turn inactivated signaling proteins (such as NF-κB and MAPKs) and endogenous antioxidants (such as Prxs, Trx, Grxs and GST) promoting apoptotic processes. When MnP is combined with exogenous sources of H_2_O_2_, such as chemo- and radio-therapy as well as ascorbate, the effect of MnP on tumor growth suppression is further enhanced [16,18,23,25].

Several reports have emphasized the significance of the epithelial-to-mesenchymal transition (EMT) as a key step in enhancing cancer cell invasion and metastasis [26,27,28]. EMT is the reprogramming of epithelial cells to a mesenchymal-like phenotype and is mediated by a set of transcription factors such as Slug, Snail, Twist, and Zeb1/2. The transcription factors can inhibit the expression of the epithelial marker E-cadherin and induce the expression of mesenchymal markers such as N-cadherin, vimentin, and fibronectin [29,30,31]. Acquisition of migratory and invasive properties of tumor cells through the EMT process is a prerequisite for metastasis [26,32].

In a glioma mouse subcutaneous xenograft study, the gene expression analysis showed that the butoxyalkyl analog, MnTnBuOE-2-PyP^5+^, in the presence of radiation inhibits metastatic pathways (*ctss*, *cathepsin L*, *becnl*, *beclinl*) [7,18,19]. In recent studies, MnHex suppressed the migration of human kidney [33] and breast cancer cells [34]. In the present study, we went a step further and explored the MnHex-driven metastasis in both cellular and animal models focusing on the migration of tumor cells and the factors that modulate this behavior. Through understanding the molecular mechanisms involved in the inhibition of metastasis, we aimed at exploring the rationale for the progress of MnPs into clinical trials.

## 2. Materials and Methods

### 2.1. Cell Culture and Irradiation

Mouse mammary carcinoma 4T1 cells and MDA-MB-231 human triple-negative breast cancer cells were obtained from the Korean Cell Line Bank (Seoul National University, Seoul, Korea), and cultured in RPMI-1640 medium supplemented with 10% fetal bovine serum (FBS), 2 mM L-glutamine, 100 U/mL penicillin, 100 g/mL streptomycin, and 25 mM HEPES (Gibco, Carlsbad, CA, USA). Cultures were maintained in a humidified atmosphere of 95% air/5% CO_2_ at 37 °C. The luciferase-expressing 4T1-Red-Fluc mouse breast cancer cell line was obtained from Perkin Elmer (Waltham, MA, USA) and was grown in RPMI-1640 medium without antibiotics, as recommended by the supplier. All animal procedures were conducted in accordance with appropriate regulatory standards under protocol (ID: 20170718001; approval date: 18 July 2017 and ID: 20181228001; approval date: 31 December 2018) approved by the Institutional Animal Care and Use Committee (IACUC) of the Samsung Biomedical Research Institute (SBRI) at Samsung Medical Center (SMC).

For RT treatment, cell monolayers were irradiated with various doses of 6 MV photons at a dose rate of 3.96 Gy/min using a Varian Clinac 6EX linear accelerator (Varian Medical Systems, Palo Alto, CA, USA).

### 2.2. Reagents and Antibodies

The Mn porphyrin-based SOD mimic, MnTnHex-2-PyP^5+^ (MnHex) (λmax = 454.5 nm, log ε = 5.21), was obtained from Ines Batinic-Haberle at Duke University School of Medicine. Transwell and Matrigel were from Corning (Beverly, MA, USA). Antibodies against E-cadherin, N-cadherin, Snail, Twist, STAT3, p-STAT3, PARP, AKT, p-AKT, GSK3β, p-GSK3β, and HO-1 were from Cell Signaling Technology (Beverly, MA, USA). Anti-NRF2 antibodies were from Santa Cruz Biotechnology (Dallas, TX, USA). Anti-β-actin, anti-rabbit IgG, and anti-mouse IgG secondary antibodies were from Sigma-Aldrich (St. Louis, MO, USA). Neutral buffered formalin (NBF; 10%) was from Sigma-Aldrich. Anti-ZO-1 antibodies and Alexa-Fluor 488-conjugated secondary antibodies were from Life Technologies (Eugene, OR, USA). Unless otherwise noted, all other chemicals were from Sigma-Aldrich.

### 2.3. Wound-Healing Assays

4T1 and MDA-MB-231 cells were seeded at 1 × 10^6^ cells per well in 6-well plates. At 90% confluence, the cells were pretreated with MnHex (2 μM) or sham (PBS) for 4 h, followed by exposure to radiation (0 and 4 Gy). Immediately after irradiation, the monolayer was scratched with a sterile pipette tip and washed by phosphate-buffered saline (PBS) to eliminate the impaired cells. The medium was replaced with fresh medium without FBS. The wound area was measured using ImageJ software version 1.53e (National Institutes of Health, Bethesda, MD, USA). The wound area percentage was calculated as the ratio of wound area at 48 h to the wound area at 0 h.

### 2.4. Transwell Migration Assay/Invasion Assay

Cells (1 × 10^4^ cells per well) were plated in the upper chambers of Transwell plates (24-well insert, pore size: 8 mm) in medium without FBS. Medium with FBS was added to the lower chambers and, 24 h later, the cells were pretreated with MnHex (2 μM) or sham (PBS) for 4 h, followed by exposure to radiation (0 and 4 Gy). After incubation at 37 °C for 72 h, non-migrated cells on the upper membrane surface were wiped off with a cotton swab. Cells that had migrated to the lower surfaces of each filter were fixed with 10% neutral buffered formalin solution for 30 min and stained with 0.05% crystal violet solution for 10 min.

For the invasion assay, 5 × 10^4^ cells were plated in the top chamber on a Matrigel-coated membrane. Medium without serum was added to the upper chamber, and medium containing 10% FBS was added to the lower chamber.

### 2.5. Western Blotting

Cells were harvested, washed with PBS, and lysed in 20 mM Tris (pH 8.0), 137 mM NaCl, 10% glycerol, 1% Nonidet P-40, 10 mM EDTA, 100 mM NaF, 1 mM phenylmethylsulfonyl fluoride, and 10 mg/mL leupeptin. Lysates were centrifuged at 15,000× *g* for 15 min, and the concentration of protein in each lysate was determined using Bio-Rad protein assay reagent (Bio-Rad Laboratories, Hercules, CA, USA), according to the manufacturer’s recommendations. Thereafter, 8%, 10%, or 12% sodium dodecyl sulphate–polyacrylamide gel electrophoresis was used to separate 20 μg protein samples. Following electrophoresis, proteins were transferred to nitrocellulose membranes (Bio-Rad Laboratories), blocked overnight in 5% skim milk in PBS at 4 °C, and subsequently probed with a primary antibody. The blots were also probed with a monoclonal anti-β-actin antibody (Sigma-Aldrich) to be quantified as a reference loading control. The detection of specific proteins was carried out with enhanced chemiluminescence detection reagents (GE Healthcare, Marlborough, MA, USA) following the manufacturer’s instructions. Representative images from at least two independent experiments are shown. Relative band intensity was quantified using ImageJ and normalized to β-actin band intensity.

### 2.6. Immunofluorescence

Cells (2 × 10^4^) were cultured on cover glasses (Paul Marienfeld GmbH & Co. KG, Lauda-Königshofen, Germany) in a 12-well plate, fixed with 4% formaldehyde and permeabilized with 0.01% Triton X-100. After blocking with 2% FBS for 30 min, cells were incubated with primary antibody for 1 h, followed by DAPI (Sigma-Aldrich), Alexa Fluor-488-conjugated secondary antibody, and Alexa Fluor488-conjugated or Alexa Fluor555-conjugated phalloidin (Life Technologies) for 30 min. Cells were then washed, mounted using fluorescent mounting medium (Dako, Carpinteria, CA, USA), and analyzed using a fluorescence microscope (Zeiss Observer D1; Carl Zeiss Co., Ltd. Carpinteria, CA, USA).

### 2.7. Transfection

Small interfering RNA (siRNA) was used to knock down the Nrf2 protein in 4T1 cells. For transfection, 7 × 10^5^ 4T1 cells were seeded onto 100 mm dishes and incubated with a mixture of 10 nM siRNA and Lipofectamine RNAiMAX (1:1.5 ratio; Thermo Fisher Scientific, Waltham, MA, USA) for 16 h. NRF2-targeting siRNA (sc-37049) and control siRNA (sc-37007) were purchased from Santa Cruz Biotechnology (Santa Cruz, CA, USA). Knockdown of NRF2 was confirmed using Western blotting.

### 2.8. Animal Models

For the 4T1 tumor model, 6–7-week-old female BALB/c mice were purchased from Orient Bio (Gapyeong, Korea). Cells (1 × 10^5^ cells in 50 µL PBS) were injected subcutaneously into the right hind leg. Tumor volumes were measured every 3 days using calipers and calculated as volume = (width^2^ × length)/2. When the mean tumor volume reached 80–120 mm^3^, mice were randomized into four groups. MnHex (1 mg/kg) was administered by intraperitoneal injection every 3 days starting on the day of randomization. The injections were continued until the day prior to euthanasia. Two hours after drug treatment, the tumor was irradiated with 2 Gy X-rays for three consecutive days for a total of 6 Gy to the right hind leg. During irradiation, the mice were anesthetized by intraperitoneal injection of 30 mg/kg Zoletil (Virbac, Carros, France) and 10 mg/kg Rompun (Bayer, Leverkusen, Germany) as prescribed by veterinarians.

For the lung metastatic tumor model, 24 h pre-irradiated (6 Gy in three fractions) or non-irradiated 4T1-Red-Fluc cells (1 × 10^4^ cells in 100 µL PBS) were injected into the BALB/c mice through the tail vein. MnHex (1 mg/kg) was administered by intraperitoneal injection every 3 days. Injections were continued until the day prior to euthanasia.

For the MDA-MB-231 metastatic tumor model, 24 h pre-irradiated (6 Gy in three fractions) or non-irradiated MDA-MB-231 cells (1 × 10^5^ cells in 100 µL PBS) were injected into the BALB/C nude mice through the tail vein.

Animals were housed under barrier conditions at 21 °C with a 12/12 h light/dark cycle and fed a standard rodent diet and water.

### 2.9. In Vivo Bioluminescence

Images were obtained with the approval and technical support of the Molecular and Cellular Imaging Center of Samsung Biomedical Research Institute. Labeled 4T1 cells were assessed weekly from 1 to 7 weeks after cell injection using an in vivo bioluminescence imaging system. For IVIS imaging, each mouse was intraperitoneally injected with 150 mg/kg D-luciferin (PerkinElmer, Waltham, MA, USA). Mice were anesthetized with a mixture of 1.5% isoflurane/air using an inhalation anesthesia system (VetEquip, Inc., Pleasant Hill, CA, USA). Ten minutes after D-luciferin injection, mice were imaged using an IVIS Spectrum In Vivo Imaging System (PerkinElmer) with continuous 1% isoflurane/air maintenance. Imaging data were analyzed using OptixMX3 software (Advanced Research Technologies Inc., Saint-Laurent, QC, Canada).

### 2.10. Immunohistochemistry

Necropsies were performed after euthanasia and organs were immediately placed into 10% neutral-buffered formalin (NBF). Tissues were processed and embedded in paraffin and sectioned at 4 μm. Sections were stained with hematoxylin and eosin (H&E) for routine histological evaluation. Following H&E staining, tissues were stained with the primary antibodies against Snail, E-cadherin, and N-cadherin. Slides were digitally scanned with a digital pathology scanning system (Aperio ScanScope AT; Leica Biosystems, Buffalo Grove, IL, USA) and analyzed using ImageScope software (Leica Biosystems).

### 2.11. Statistical Analysis

Multiple groups were compared using one-way analysis of variance (ANOVA) with Tukey’s multiple comparison test using GraphPad Prism 8 (GraphPad Software, San Diego, CA, USA). Tumor growth curves were analyzed using a two-way analysis of variance (ANOVA) with Tukey’s correction for multiple comparisons. The statistical details of each experiment are indicated in the figure legends.

## 3. Results

### 3.1. Radiation Induces the Changes of Morphology and the Expression of EMT-Related Proteins in 4T1 Cells

**Figure 1:**
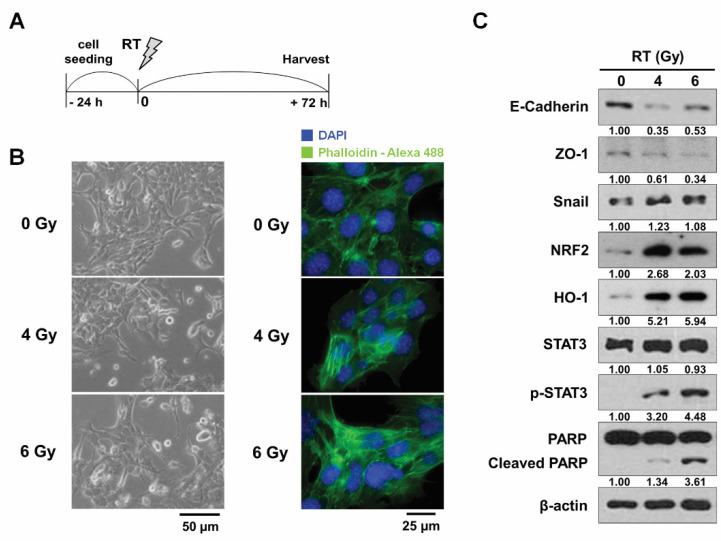
**Radiation promotes EMT in 4T1 cells.** (**A**) Scheme of radiation treatments for in vitro experiments. (**B**) Representative phase-contrast micrographs (**left**) and immunofluorescence micrographs (**right**) depicting morphological changes caused in 4T1 cells by radiation treatment. The cells were imaged 72 h after X-ray irradiation. (**C**) Radiation treatment-induced changes in EMT marker expression by Western blotting. Cells were collected 72 h after irradiation. β-actin was used as a loading control. RT, radiation therapy.

At first, to confirm the change of the metastatic potential of breast cancer cells by RT, we observed morphological changes of mouse mammary carcinoma 4T1 cells 72 h after irradiation (Figure 1A). The unirradiated 4T1 cells had a classical epithelial morphology, and the cells irradiated with single doses of 4 and 6 Gy X-rays displayed an elongated spindle-like morphology with an actin cytoskeleton rearranged into stress fibers (Figure 1B).

Next, we determined the effects of RT on the expression of EMT-related proteins in 4T1 cells. Western blotting showed that RT treatment significantly reduced expression of epithelial markers, such as E-cadherin and tight junction protein ZO-1, and triggered the expression of Snail, an EMT regulator (Figure 1C). Phosphorylation of STAT3 and cleavage of PARP, which are markers for EMT and apoptosis, respectively, were also promoted by 6 Gy radiation. The expression of NRF2 and its downstream HO-1 was also stimulated by RT treatment (Figure 1C). Together, our data demonstrated that RT induced EMT and apoptosis in 4T1 cells.

### 3.2. Co-Treatment with Radiation and MnHex Inhibits Migration and Invasion of 4T1 Cells In Vitro

**Figure 2:**
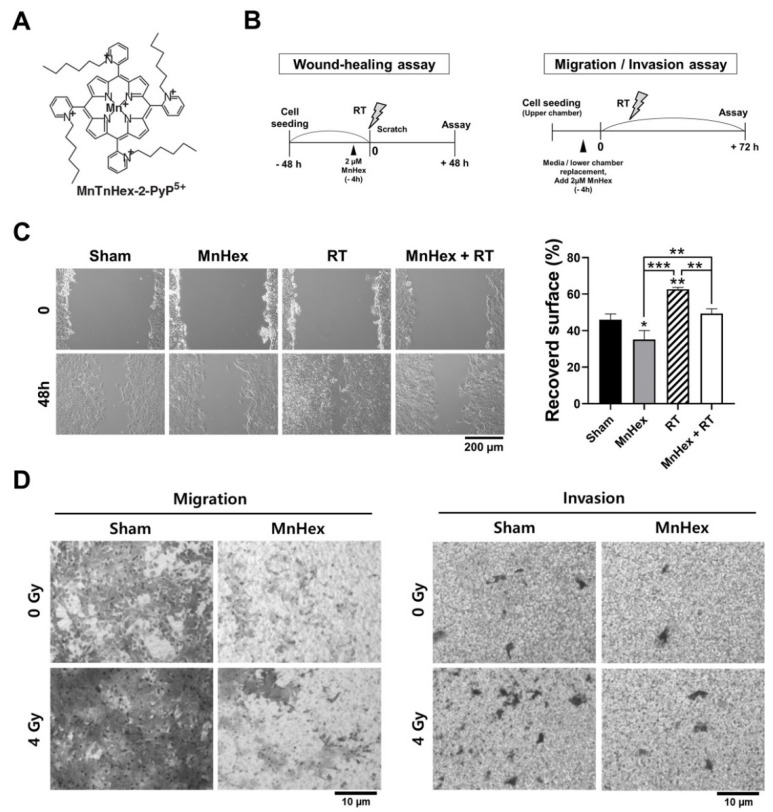
**Co-treatment with MnHex and radiation inhibits migration and invasion of 4T1 cells.** (**A**) Chemical structure of MnHex used in this study. (**B**) Schemes for MnHex and RT treatment for wound-healing (**left**) and migration/invasion (**right**) assays. (**C**) Wound-healing assay showing inhibition of cell migration by MnHex/RT co-treatment. Representative micrographs (**left**) and quantification (**right**) are presented. Recovered surface was calculated as percentage of wound closure area between 0 and 48 h after scratch. Data are presented as the mean ± SD (*n* = 3); * *p* < 0.05; ** *p* < 0.01; *** *p* < 0.001. (**D**) Transwell migration and invasion assays showing that MnHex inhibited RT-induced migration and invasion.

The biological effects of RT on 4T1 breast cancer cells and the inhibitory effect of MnHex/RT co-treatment on cell migration and invasion were measured using wound-healing and Transwell migration/invasion assays (Figure 2A,B). The wound-healing assay showed that compared with the control, RT significantly promoted the recovery of wounds (Figure 2C, *p* < 0.01), which was suppressed by MnHex pretreatment (*p* < 0.01). To further determine the effect of MnHex on migration and invasion, 4T1 cells were seeded into Transwell inserts with and without Matrigel coating, and then the cells that migrated through the membrane were stained. These assays showed that RT-induced migration and invasion were both reduced by MnHex pretreatment (Figure 2D). These data demonstrate that MnHex plays an inhibitory role in RT-induced migratory phenotypes related to metastasis.

### 3.3. MnHex Suppresses Radiation-Induced EMT Signaling in 4T1 Cells

**Figure 3:**
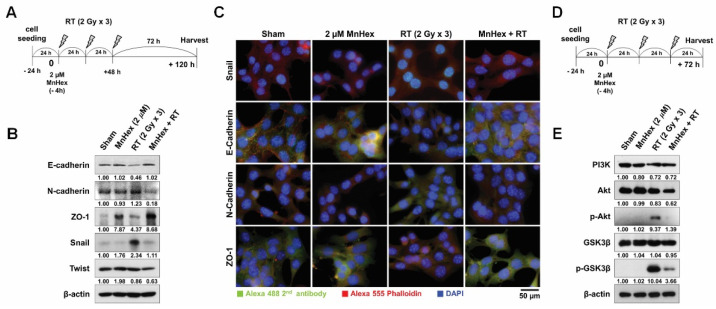
**MnHex suppresses radiation-induced EMT in 4T1 cells.** (**A**) Scheme for the fractionated irradiation and MnHex treatment for determining late response. (**B**) MnHex inhibited the expression of radiation-induced EMT markers in 4T1 cells, which was evaluated by using Western blotting. β-actin was used as a loading control. (**C**) Representative immunofluorescence micrographs of anti-Snail, anti-E-cadherin, anti-N-cadherin, and anti-ZO-1 staining. (**D**) Scheme for fractionated irradiation and MnHex treatment for determining early response. (**E**) MnHex inhibited radiation-mediated AKT/GSK3β signaling. β-actin was used as a loading control.

To test whether the reduction of migration by MnHex and RT is related to the EMT phenotype, we determined changes in the expression of EMT-related proteins using Western blotting and immunofluorescence. The 4T1 cells were pretreated with MnHex for 4 h and then irradiated with 6 Gy in three fractions. To investigate the late response to RT, cells were harvested 72 h after irradiation (Figure 3A). While fractionated RT suppressed the expression of E-cadherin, it promoted that of N-cadherin and Snail but not Twist, as determined by Western blot analysis (Figure 3B). Pretreatment with MnHex reversed the RT-mediated changes in the expression of EMT-related proteins (Figure 3B). The expression of ZO-1, a tight junction protein, was augmented by MnHex treatment. Immunofluorescence staining for Snail, E-cadherin, N-cadherin, and ZO-1 confirmed that MnHex blocked RT-induced EMT (Figure 3C).

To understand the effects of MnHex on early signaling events after RT, 4T1 cells were collected 24 h after fractionated RT (Figure 3D). Fractionated RT promoted phosphorylation of AKT, followed by an increase in inactive phosphorylation of GSK3β, both of which were suppressed by MnHex pretreatment (Figure 3E). Subcellular fractionation revealed that MnHex upregulated the expression of HO-1 and NRF2; for NRF2, an increase was seen in both cytosolic and nuclear fractions (Figure S1A). Immunofluorescence also confirmed that nuclear enrichment of NRF2 was enhanced by MnHex (Figure S1B).

To determine whether MnHex-activated NRF2 signaling contributes to the anti-metastatic effect of MnHex, we depleted NRF2 using siRNA treatment and then tested the effect of MnHex and RT on cell migration and invasion (Figure S2). Western blotting showed that NRF2 expression was reduced in 4T1 cells transfected with NRF2 siRNA, compared to those transfected with non-targeting control siRNA (Figure S2A). The wound-healing assay showed that MnHex pretreatment inhibited RT-induced cell migration while NRF2 knockdown enhanced wound closure in all experimental conditions (Figure S2B). Similarly, the Transwell assay showed that RT stimulated cell migration and invasion, which was further enhanced by NRF2 knockdown (Figure S2C,D). Yet, MnHex pretreatment inhibited RT-induced cell migration and invasion to a similar degree in both control and NRF2 knockdown 4T1 cells (Figure S2B–D). The knockdown data suggest that NRF2 has a suppressive role in cell migration and invasion, and its activation by MnHex contributes to its anti-metastatic potential. Further studies are needed to clarify the action of Mn porphyrins in relation to NRF2.

### 3.4. Co-Treatment with Radiation and MnHex Inhibits Lung Metastasis of 4T1 Tumors In Vivo

**Figure 4:**
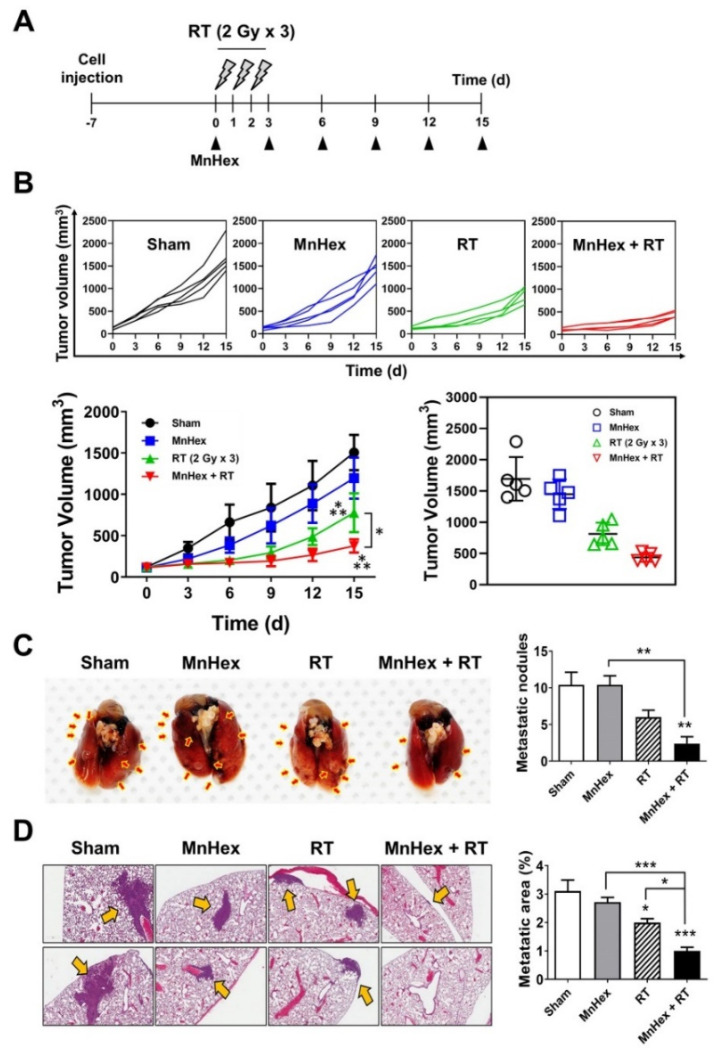
**Co-treatment with MnHex and radiation inhibits tumor growth and spontaneous lung metastasis in a 4T1 tumor mouse model.** (**A**) Scheme for radiation and MnHex treatment in mice bearing 4T1 tumors. Once tumors were palpable, they were irradiated with 6 Gy in three fractions. Mice were intraperitoneally given 1 mg/kg/day of MnHex every 3 days. (**B**) MnHex enhanced radiation-induced tumor growth delay. BALB/c mice bearing 4T1 tumors were treated with vehicle (PBS), MnHex, radiation or MnHex/RT co-treatment. Tumor growth curves of individual mice (upper panels), averaged tumor growth curves (lower left panel) and tumor volumes at day 15 (lower right panel) are shown. Data are presented as the means ± SD (*n* = 5); * *p* < 0.05; *** *p* < 0.001. (**C**) Representative photographs of lungs from 4T1 tumor mice (left panel). Red arrows indicate metastatic nodules. The graphical data represent the count of metastatic nodules per lung (right panel). (**D**) Representative H&E images of lung tissues showing metastatic foci as indicated by yellow arrows (right panel). Metastatic area was quantified by calculating the area covered by tumor mass relative to the total area of lung tissue sections (*n* = 5). For all graphs, data are presented as the mean ± SD; * *p* < 0.05; ** *p* < 0.01; *** *p* < 0.001.

Next, we determined whether suppression of EMT by MnHex/RT co-treatment decreased metastasis in vivo using the 4T1 tumor-bearing BALB/c mice model. The 4T1 tumors were generated by subcutaneously inoculating cells into the right hind leg and then irradiated with 6 Gy in three fractions once the tumors were palpable (Figure 4A). Fractionated RT significantly inhibited tumor growth (*p* < 0.001); moreover, its combination with MnHex further suppressed tumor growth (*p* < 0.01; Figure 4B). Spontaneous metastasis of 4T1 cells to lungs was evaluated by counting metastatic nodules on the lung surface (Figure 4C) and hematoxylin and eosin (H&E) staining of lung tissues (Figure 4D). Fractionated RT but not MnHex alone inhibited lung metastasis; this inhibition was significantly enhanced by co-treatment (Figure 4C,D). Together, our data suggest that MnHex/RT co-treatment inhibits spontaneous lung metastasis of 4T1 cells implanted into hind legs in vivo.

**Figure 5:**
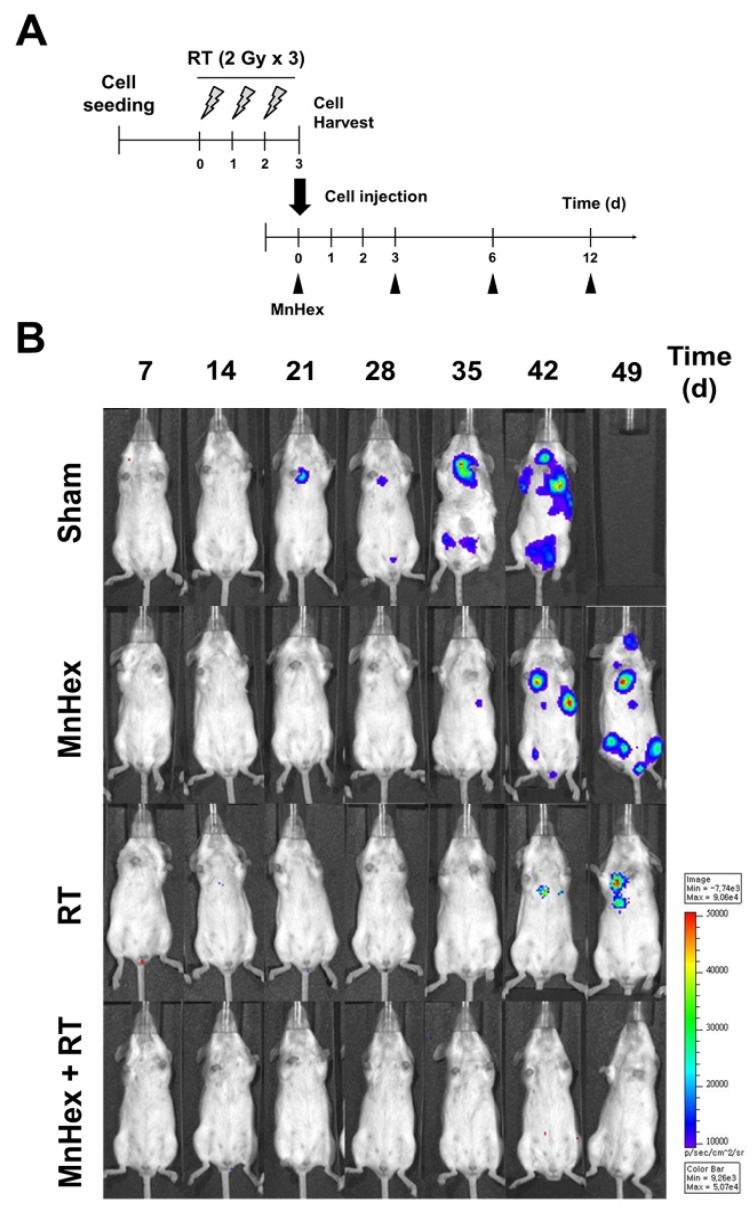
**MnHex inhibits lung metastasis of 4T1 cells in an intravenous injection model.** (**A**) Scheme for radiation and MnHex treatment for a metastasis mouse model. The 4T1 cells were irradiated with 6 Gy in three fractions and then were intravenously injected into BALB/c mice. Mice were treated with 1 mg/kg of MnHex three times per week for 12 days via intraperitoneal injection. (**B**) IVIS imaging showed that MnHex/RT co-treatment inhibited lung metastasis after tail vein injection of luciferase-labeled 4T1 cells.

As another metastasis model, we injected luciferase-labeled 4T1 cells into the tail veins of BALB/c mice and determined lung metastasis using non-invasive bioluminescence imaging. Before tail-vein injection, 4T1 cells were irradiated with 6 Gy in three fractions and then harvested. Sham-irradiated cells were used as controls. Identical numbers of viable cells were intravenously injected into mice, followed by intraperitoneal MnHex injection three times per week (Figure 5A). Bioluminescence imaging revealed that MnHex/RT co-treatment dramatically inhibited lung metastasis of 4T1 cells in BALB/c mice (Figure 5B).

**Figure 6:**
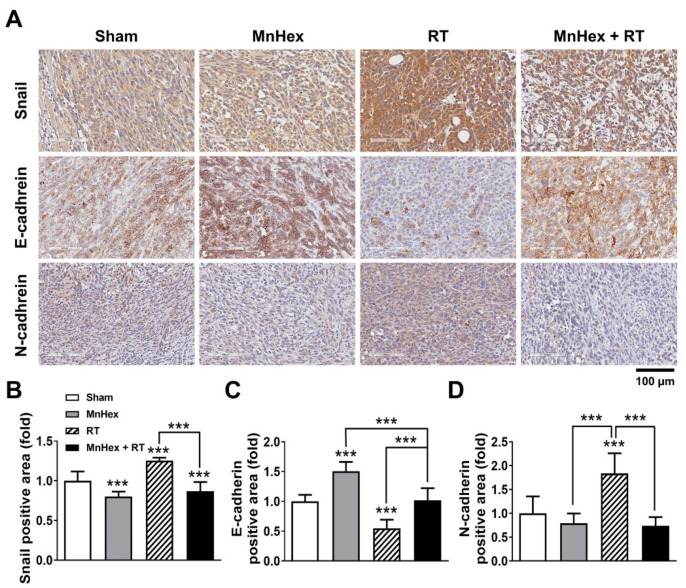
**MnHex inhibits radiation-induced expression of EMT markers in 4T1 tumor tissues.** (**A**) Representative IHC staining images of Snail, E-cadherin, and N-cadherin. 4T1 tumors were harvested from mice that received radiation, MnHex, or the MnHex/RT co-treatment on day 15. Paraffin-embedded tissues sections were stained as described in Materials and Methods. (**B**–**D**) Quantification data of Snail (**B**), E-cadherin (**C**), and N-cadherin (**D**) in 4T1 tumor tissues. For all graphs, data are presented as mean ± SD (*n* ≥ 12); *** *p* < 0.001.

To test whether the inhibition of lung metastasis by MnHex/RT is related to the EMT, we performed immunohistochemistry (IHC) staining of 4T1 tumor tissues for EMT markers, including N-cadherin, E-cadherin, and Snail (Figure 6A). Quantification data showed that NRF2 levels were upregulated in the mice treated with MnHex or RT compared with sham control (*p* < 0.001) and was further enhanced by co-treatment (Figure S3), which is consistent with in vitro data (Figure S1B). RT treatment decreased the expression of E-cadherin and increased that of N-cadherin and Snail (all *p* < 0.001) in 4T1 tumor tissues compared with sham controls, which was reversed by MnHex/RT co-treatment (Figure 6B–D). These data are consistent with our in vitro data shown in Figure 3B,C, demonstrating that MnHex/RT suppresses spontaneous metastasis of 4T1 cells by blocking EMT.

### 3.5. Co-Treatment with Radiation and MnHex Inhibits Metastatic Potential of MDA-MB-231 Cells In Vitro and In Vivo

**Figure 7:**
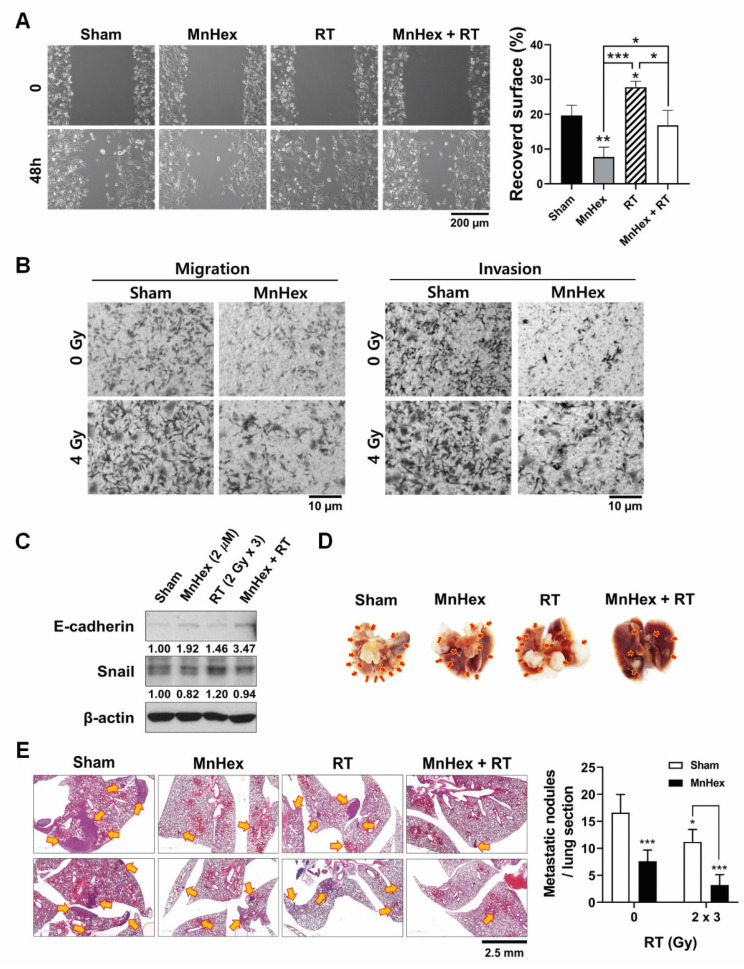
**Co-treatment with MnHex and radiation inhibits metastatic potential of MDA-MB-231 in vitro and in vivo.** (**A**,**B**) Suppression of migration and invasion of MDA-MB-231 cells by MnHex/RT co-treatment assessed by (**A**) wound-healing and (**B**) Transwell migration and invasion assays. The schedule of radiation and MnHex treatment was the same as described in Figure 2B. (**C**) Western blotting showed MnHex suppressed radiation-induced EMT marker expression in MDA-MB-231 cells. (**D**) Representative micrographs showing lung metastases after tail vein injection of MDA-MB-231 cells. The schedule of radiation and MnHex treatment was as described in Figure 5A. Red arrows indicate metastatic nodules. (**E**) Representative H&E micrographs of lung tissues. Yellow arrows indicate metastatic foci. Right, counts of metastatic nodules per lung tissue sections (*n* = 5). For all graphs, data are presented as the mean ± SD; * *p* < 0.05; ** *p* < 0.01; *** *p* < 0.001.

Based on the findings from the in vitro and in vivo studies using 4T1 cells, we tested whether MnHex inhibits the metastatic potential of human triple-negative breast cancer MDA-MB-231 cells. When treatment schedules similar to those for 4T1 cells were applied, RT-induced cell migration and invasion were suppressed by MnHex pretreatment in the MDA-MB-231 cells (Figure 7A,B). Although MDA-MB-231 cells had mesenchymal phenotypes, the expression of Snail was further induced by fractionated RT and was suppressed by MnHex pretreatment (Figure 7C). The level of E-cadherin was very low in MDA-MB-231 cells but was augmented by MnHex treatment. Together, our data demonstrate that MnHex reversed EMT in MDA-MB-231 cells in vitro. Similarly, MnHex treatment suppressed RT-induced expression of the mesenchymal markers, fibronectin, α-smooth muscle actin (αSMA), and Snail in MCF7, a luminal type human breast cancer cell line (Figure S4).

Next, we tested whether administration of MnHex inhibits lung metastasis of MDA-MB-231 cells. For this experiment, we injected MDA-MB-231 cells irradiated with 6 Gy in three fractions into the tail vein of BALB/c nude mice. This was followed by intraperitoneal injection of MnHex. Two weeks after cell injection, the mice were euthanized, and lung tissues were excised. Metastatic nodule counts decreased by co-treatment with MnHex/RT, compared with the sham controls and both single treatments (Figure 7D). Counting of metastatic nodules in the H&E-stained lung tissues also confirmed that co-treatment with MnHex/RT effectively suppressed lung metastasis of MDA-MB-231 (Figure 7E). Thus, these findings demonstrate that MnHex suppressed metastatic potential of mesenchymal MDA-MB-231 cells as well as 4T1 cells retaining epithelial characteristics.

## 4. Discussion

While the direct cytotoxic effects of radiation on cells and tissues are well known, local treatment of primary tumors with radiation also has other unpredictable systemic effects on tumor growth, such as enhanced growth of distant metastases as well as inhibition of distant tumor growth, also known as the abscopal effect. However, the relevance of these effects to clinical experience and the mechanisms involved remains unclear. It is important to understand both local and systemic effects induced or influenced by radiation to minimize recurrences and other adverse effects while optimizing tumor control. Metastasis occurs through the acquisition of an invasive, migratory phenotype by cancer cells, leading to invasion into local tissues and subsequent entry into the circulation and trafficking to distant sites [32]. Thus, migration of tumor cells is a prerequisite for both invasion and metastasis. In particular, radiation can affect alterations in tumor cells themselves, changes in the microenvironment, and interactions between them.

Several reports have emphasized the significance of the EMT as a key step in enhancing cancer cell invasion and metastasis [26,27,28]. EMT is mediated by transcription factors, such as Slug, Snail, Twist, and Zeb1/2, which can inhibit the expression of the epithelial marker E-cadherin and induce the expression of mesenchymal markers, including N-cadherin, vimentin, and fibronectin. EMT is triggered by a variety of signaling pathways, among which the transforming growth factor β (TGF-β) signaling pathway has been implicated as a primary inducer. Reportedly, EMT is also under the control of the NRF2 transcription factor [35].

We have previously reported that MnTE-2-PyP^5+^ and MnTnHex-2-PyP^5+^ downregulated TGF-*β* expression in a rat model of pulmonary radioprotection [36,37,38]. Recently, Yu et al. [28] showed that MnTE-2-PyP^5+^ treatment reversed cell phenotypes induced by TGF-*β* in colon cancer cells and significantly reduced the expression of mesenchymal markers but maintained epithelial marker expression. Oberley-Deegan’s team showed that MnTE-2-PyP^5+^ suppressed the phosphorylated Smad2/3 protein levels, induced by TGF-*β* in SW480 cells, but it failed to suppress TGF-*β*-induced Slug and Snail expression in colorectal cells [30]. Furthermore, MnTE-2-PyP^5+^ effectively suppressed TGF-*β*-mediated cell migration and invasion and the expression of matrix metalloproteinases 2 and 9 in colorectal cancer [30]. Finally, Fernandes’s team reported the ability of MnTnHex-2-PyP^5+^ to enhance the cytotoxicity of doxorubicin to MDA-MB-231 and MCF-7 breast cancer cells, via increasing H_2_O_2_ levels, and reducing collective cell migration and chemotaxis thereby affecting their metastatic potential [34].

**Figure 8:**
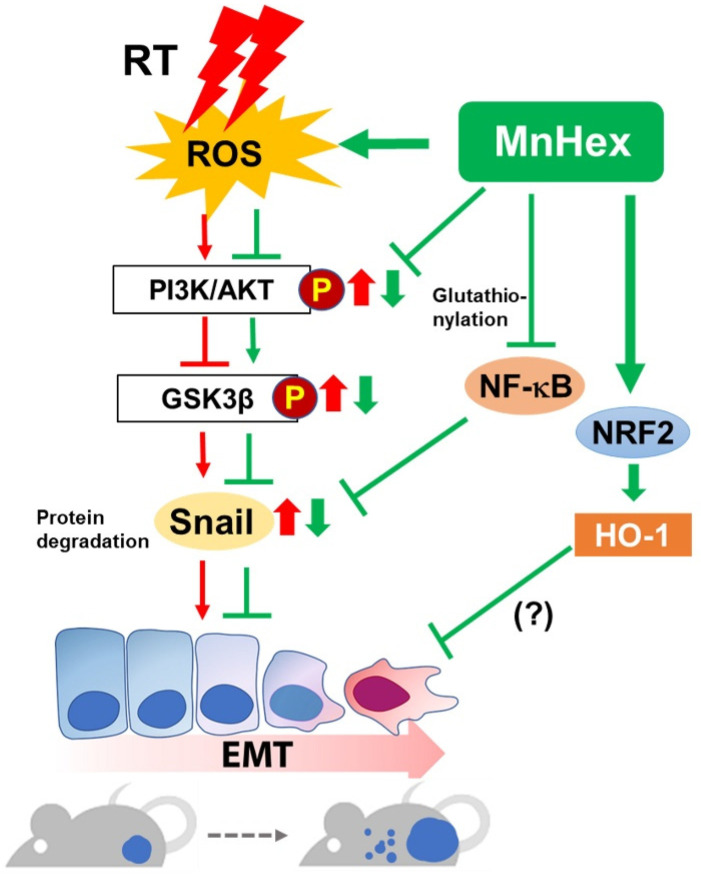
**Schematic illustration of the proposed mechanism for the anti-metastatic effect of MnHex in breast cancer models.** Our study reveals that MnHex inhibits RT-induced EMT via blocking AKT/GSK3β/Snail pathway, thereby exerting an anti-metastasis effect in mouse models. RT-induced ROS activate AKT, which phosphorylates GSK3β. Inactivation of GSK3β blocks degradation of Snail, inducing EMT. MnHex reverses these phenomena, which may diminish metastatic potential of RT-treated cancerous cells. While not investigated here, the reported data show that Snail is under the control of NF-кB signaling the latter reported to be inactivated by MnHex. MnHex, MnTnHex-2-PyP^5+^; RT, radiation therapy; ROS, reactive oxygen species; PI3K, phosphoinositide 3-kinase; GSK3β, glycogen synthase kinase 3 beta; EMT, epithelial-mesenchymal transition. P means a phosphorylated form of the protein. Red and green arrows indicate radiation- and MnHex-induced signaling, respectively.

In the current study, we confirmed the effect of Mn porphyrin/RT treatment on the suppression of tumor growth in the 4T1 mouse breast cancer model (Figure 4B). This is consistent with our previous work [10]; in which combined with RT, MnHex inhibited long-term colony survival and in vivo tumor growth via attenuation of DNA damage repair and induction of apoptosis, likely due to disturbed ROS balance and suppressed NF-κB signaling. We then showed that MnHex/RT treatment markedly reduced the migration of 4T1 tumor cells compared with control and RT groups (Figure 2C). In addition, our in vivo data showed that MnHex/RT co-treatment dramatically reduced lung metastasis compared with the control and each of the single treatment groups (Figure 4C,D). Mechanistically, MnHex inactivated AKT/GSK-3β/Snail pathway (Figure 3B,E) and activated NRF2/HO-1 signaling in 4T1 cells (Figure S1) which jointly led to the inhibition of EMT, resulting in diminished cell migration and invasion. Such data are in agreement with a study where the AKT/GSK3β/Snail signaling was blocked by *N*-acetylcysteine [39] and a report on the lung fibrosis where activation of NRF2 inhibits the EMT by suppressing Snail expression [35]. Finally, MnHex/RT co-treatment downregulated Snail expression (Figure 6) and upregulated NRF2 expression in 4T1 tumor tissues (Figure S3), which was accompanied by the suppressed lung metastasis in both spontaneous metastasis model (Figure 4C,D) and tail-vein injection model (Figure 5). Figure 8 depicts the actions of MnHex and RT upon the metabolic pathways involved in metastases.

The effect of MnHex on Snail, in the presence or absence of RT, might have occurred via NF-кB master transcriptional activity. Snail is a key protein in charge of AKT-induced EMT in squamous cell carcinoma cells [40] and ROS-induced EMT in breast cancer [41], which are NF-κB-dependent. Snail stabilization by NF-κB activation is also necessary for tumor necrosis factor-α (TNFα)-induced cell migration, invasion, and metastasis [42]. The direct oxidative modification of p50 and p65 subunits of NF-κB, resulting in its inactivation, has been reported by MnPs [13,16,17]. Thus, inhibition of Snail by MnHex may be controlled by complex signaling networks including NF-κB and GSK3β.

## 5. Conclusions

Thus far, the activation of NRF2 by Mn porphyrin has been reported in normal tissue only [7,18] and explored in detail by St Clair’s team on hematopoietic progenitor stem cells [43]. Ours, however, is the first study that identifies the role of Mn porphyrin in activating NRF2 in cancer cells and tumors. Anticancer drug resistance has been often attributed to the activation of NRF2, as it reduces oxidative stress in tumor cells and promotes its proliferation [44,45]. The question thus is: why the activation of NRF2 has not significantly interfered with MnP/RT-based suppression of tumor growth? Collectively, our study, redox proteomics [13,18,23], and the reported data from Jaramillo et al. [16,17] suggest that endogenous antioxidative defenses (such as Prxs, Trx, Grx, and GST) might have been upregulated by NRF2, but were oxidized by MnP/H_2_O_2_/GSH pathway and in turn inactivated [7,11,13,18]. However, with no cysteine residues available for oxidation [46], the HO-1 would stay active and contribute to the inhibition of metastases in agreement with our study and two sets of reported data on breast cancer animal models [47,48].

It is important to note that as a single drug, MnHex inhibits Snail and metastatic nodules (Figure 6 and Figure 7); the effect was recapitulated in wound healing, invasion, and migration experiments (Figure 7 and Figure S2). RT then reverses the effect of MnHex which was reinstalled when RT was coupled with MnHex (Figure 6, Figure 7 and Figure S2). Similar effects were seen with regards to NRF2. MnHex promoted the NRF2 expression which RT suppressed. Yet, MnHex coupled to RT reversed the effect of RT, increasing the NRF2 expression (Figure S1). The effects seen with MnHex as a single drug are due to its high lipophilicity thus high accumulation in all organs [49]. Consequently, high levels of MnHex in cancer cells/tumors, along with high H_2_O_2_ levels, seem to be sufficient to oxidize Keap1 and activate NRF2 (see Introduction for details on the mechanism of protein oxidation by MnPs).

Metastasis and recurrence are important factors that determine the survival rate in cancer treatments. Understanding the molecular mechanisms of anti-metastatic effects in combination therapy may help to understand the complex biological mechanism of RT. It may be a basis for identifying factors for predicting metastasis in cancer patients and for providing an important theoretical base for the development of new radiation sensitizers. Such may be the class of Mn porphyrin-based SOD mimics that would control Snail expression and activation of NRF2 as well as inflict tumor growth inhibitory effect demonstrated in previous studies [25]. We anticipate that the insights into the anticancer and antimetastatic potentials of Mn porphyrins will support their clinical development towards the improvement of the safety and efficacy of conventional RT and would significantly alleviate the suffering of the patients by preventing recurrence and improving the cure and survival rates.

## Data Availability

Data is contained within the article or Supplementary Material.

## Supplementary Materials

The following are available online at https://www.mdpi.com/article/10.3390/antiox10111769/s1, Figure S1: MnHex activates NRF2 signaling in 4T1 cells; Figure S2: Knockdown of NRF2 enhances migration and invasion of 4T1 cells; Figure S3: Immunohistochemistry of NRF2 shows that MnHex and RT increase NRF2 expression in 4T1 xenograft tumor tissues; Figure S4: MnHex treatment suppresses RT-induced expression of mesenchymal markers in MCF7 cells.
