# Supplementary material for: MnTnHex-2-PyP5+, Coupled to Radiation, Suppresses Metastasis of 4T1 and MDA-MB-231 Breast Cancer via AKT/Snail/EMT Pathways
Source: Antioxidants (Basel). 2021 Nov 5;10(11):1769. doi: 10.3390/antiox10111769 (PMC8615021; doi:10.3390/antiox10111769)
Supplement: Supplementary file 1 [file antioxidants-10-01769-s001.zip › antioxidants-1417222-supplementary.pdf]

Supplemental Material

Title: MnTnHex-2-PyP5+, coupled to radiation, suppresses metastasis of 4T1 and MDA-MB-231 breast cancer via AKT/Snail/EMT pathways

Authors: Sung Won Shin[1,2,†], Changhoon Choi[1, †], Hakyoung Kim[3], Sohee Park[1], Shin-Yeong Kim[1], Yeeun Kim[1], Ines Batinic-Haberle[4,*] and Won Park[1,2,*]

Journal: Antioxidants

† These two authors contributed equally to this work.

* These authors contribute equally to this manuscript.

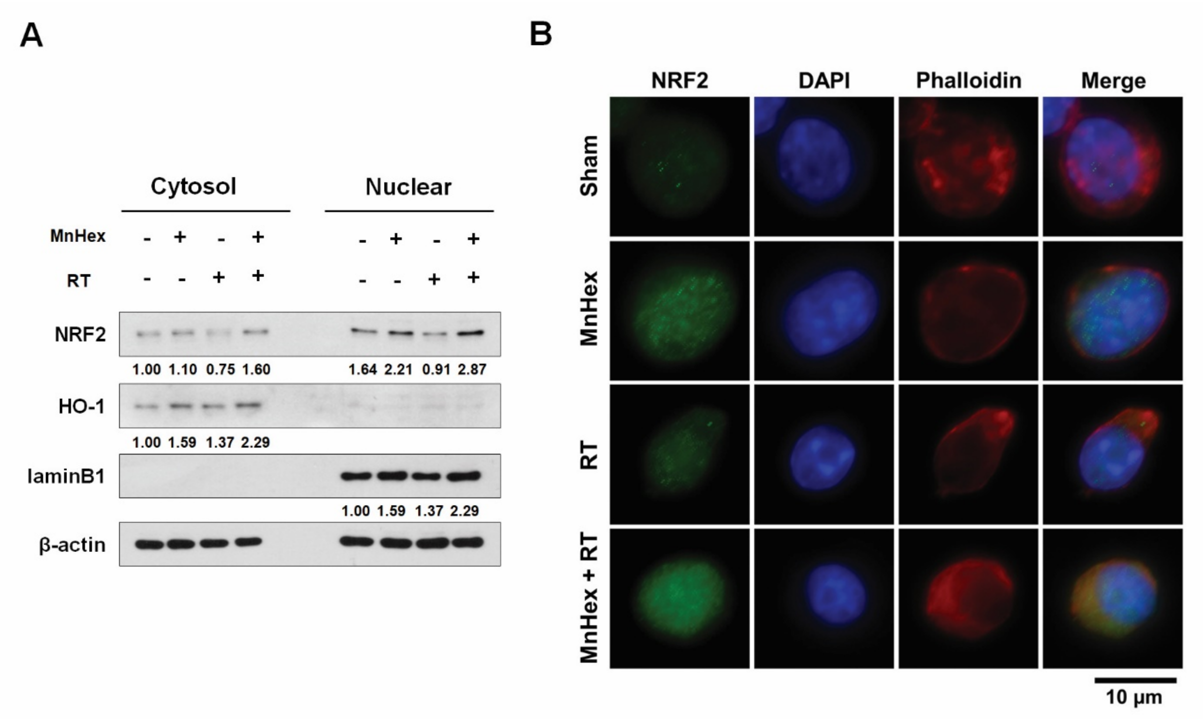

Figure S1. MnHex activates NRF2 signaling in 4T1 cells. (A) Subcellular fractionation revealed an increase in nuclear NRF2 and cytosolic HO-1 expressions by MnHex treatment. Lamin B1 was used as a nuclear marker and β-actin was used as a loading control. (B) Immunofluorescence further supported nuclear enrichment of NRF2 by MnHex treatment.

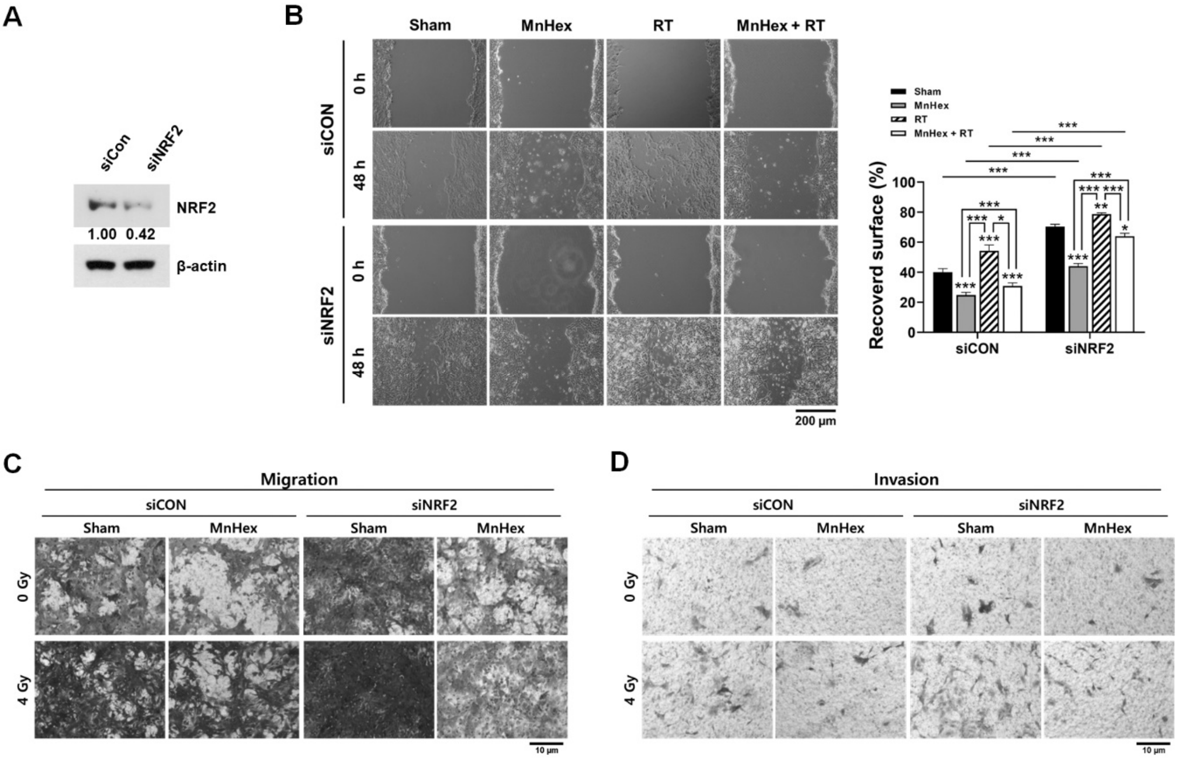

Figure S2. Knockdown of NRF2 enhances migration and invasion of 4T1 cells. (A) NRF2 knockdown was performed using siRNAs. Western blotting showed NRF2 specific siRNA (siNRF2) reduced NRF2 level, compared to control siRNA (siCon). β-actin was used as a loading control. (B) Wound-healing assay showed NRF2 knockdown increased cell migration independently of either MnHex or RT treatment. Data are presented as mean ± SD (n=3); *p<0.05; **p<0.01; ***p < 0.001. (C-D) Transwell assay showed NRF2 knockdown enhanced migration and invasion of 4T1 cells, which was enhanced by RT but suppressed by MnHex.

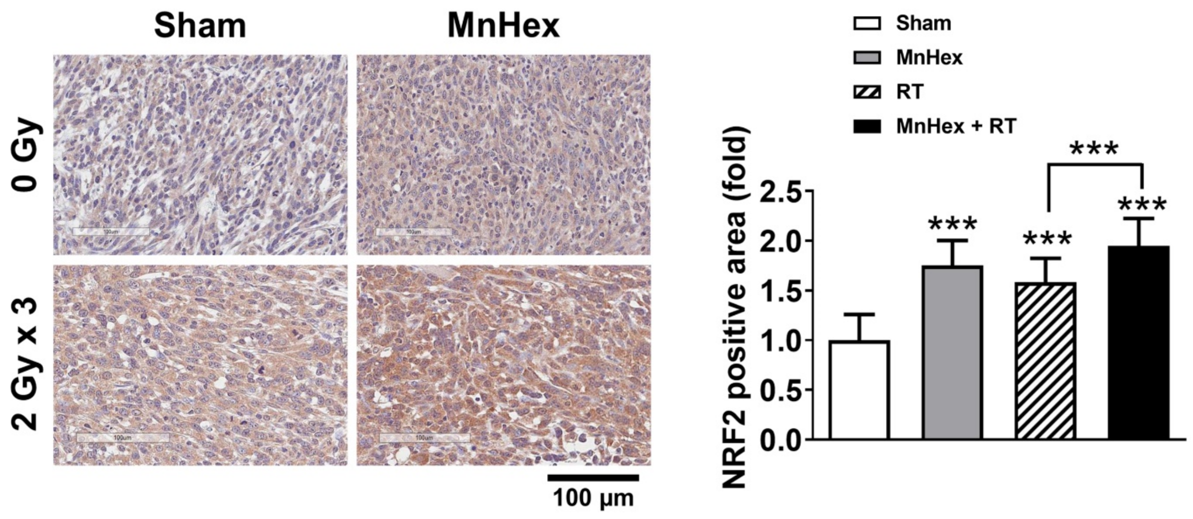

Figure S3. Immunohistochemistry of NRF2 shows that MnHex and RT increase NRF2 expression in 4T1 xenograft tumor tissues. 4T1 tumors were harvested from mice on day 15. Data are presented as mean ± SD (n=15); ***p < 0.001.

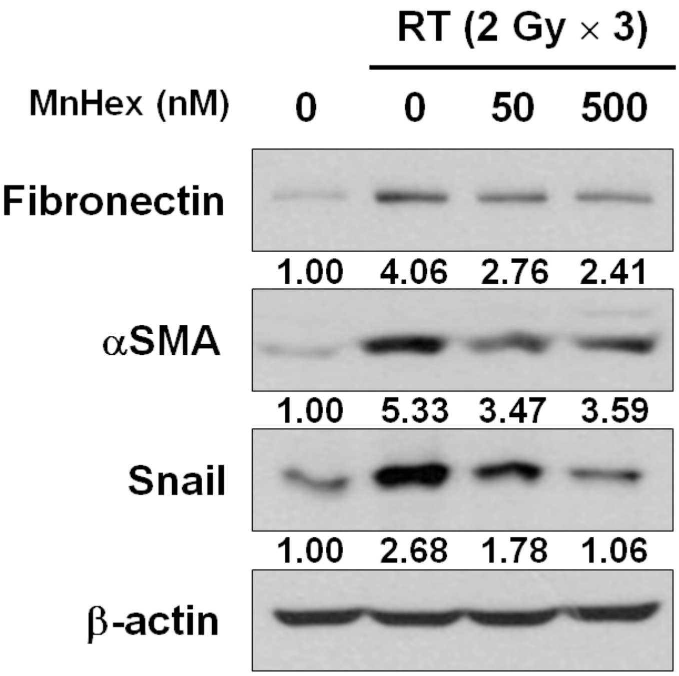

Figure S4. MnHex treatment suppresses RT-induced expression of mesenchymal markers in MCF7 cells. MCF7 cells were pre-incubated with the indicated concentrations of MnHex for 4 h, followed by fractionated RT. β-actin was used as a loading control.
